# Constructing inflammatory bowel disease diagnostic models based on k-mer and machine learning

**DOI:** 10.3389/fmicb.2025.1578005

**Published:** 2025-06-25

**Authors:** Liwei Li, Zheng Liu, Jiamin Qin, Guang Xiong, Chongze Yang, Fuqing Cai, Jiean Huang

**Affiliations:** ^1^Department of Gastroenterology, The Second Affiliated Hospital of Guangxi Medical University, Nanning, China; ^2^Department of Geriatric Endocrinology and Metabolism, The First Affiliated Hospital of Guangxi Medical University, Nanning, China; ^3^Department of Radiology, The First Affiliated Hospital of Guangxi Medical University, Nanning, China

**Keywords:** inflammatory bowel disease, gut microbiota, non-invasive diagnosis, machine learning, k-mer

## Abstract

**Background:**

Inflammatory bowel disease (IBD), encompassing Crohn’s disease (CD) and ulcerative colitis (UC), is linked to significant alterations in gut microbiota. Conventional diagnostic approaches frequently rely on invasive procedures, contributing to patient discomfort; hence, non-invasive diagnostic models present a valuable clinical alternative.

**Methods:**

Metagenomic and amplicon sequencing data were collected from fecal samples of patients with IBD and healthy individuals across diverse geographic regions. Diagnostic models were developed using Logistic Regression (LR), Support Vector Machine (SVM), Naïve Bayes (NB), and Feedforward Neural Network (FFNN), complemented by an ensemble model *via* a voting mechanism. Five-fold cross-validation facilitated the differentiation between normal controls (NC) and IBD, as well as between CD and UC.

**Results:**

K-mer-based methods leveraging metagenomic sequencing data demonstrated robust diagnostic performance, yielding ROC AUCs of 0.966 for IBD vs. NC and 0.955 for CD vs. UC. Similarly, models based on amplicon sequencing achieved ROC AUCs of 0.831 for IBD vs. NC and 0.903 for CD vs. UC. In comparison, k-mer-based approaches outperformed traditional microbiota-based models, which produced lower ROC AUCs of 0.868 for IBD vs. NC and 0.810 for CD vs. UC. Across all machine learning frameworks, the FFNN consistently attained the highest ROC AUC, underscoring its superior diagnostic performance.

**Conclusion:**

The integration of k-mer-based feature extraction with machine learning offers a non-invasive, highly accurate approach for IBD diagnosis, surpassing traditional microbiota-based models. This method holds considerable potential for clinical use, offering an effective alternative to invasive diagnostics and enhancing patient comfort.

## Graphical Abstract

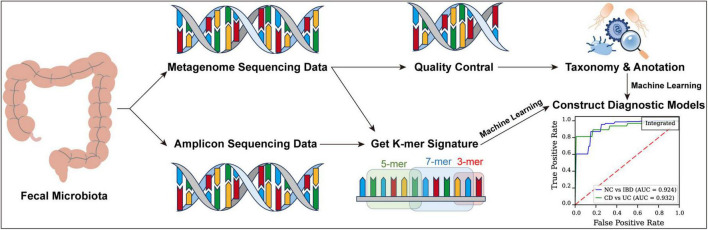

## Highlights

• Significant microbiota differences were found between NC and IBD, highlighting its role in disease progression.
• The k-mer approach outperformed traditional models, improving accuracy, especially in differentiating CD from UC.
• Using smaller k-mers (such as 3-mers and 5-mers) substantially reduces processing times for feature table generation.

## Background

Inflammatory bowel disease (IBD), comprising Crohn’s disease (CD) and ulcerative colitis (UC), is a chronic gastrointestinal disorder characterized by recurrent inflammation, which severely diminishes patient quality of life. Affecting millions globally, the highest incidence rates are observed in North America and Europe, with approximately 0.2% of the European population diagnosed (Zhao et al., 2020) and an incidence rate of 10.9 per 100,000 person-years in the United States (Lewis et al., 2023). Prolonged chronic inflammation in patients with IBD leads to complications such as intestinal strictures, fistulas, and colorectal cancer, further impacting health and well-being (Rieder et al., 2016; Shah and Itzkowitz, 2022).

Immune factors, genetic predisposition, environmental influences, and the gut microbiota are recognized as the four principal triggers of IBD. Over the past 2 decades, genome-wide association studies have identified more than 200 IBD risk genes (El Hadad et al., 2024); in these genetically susceptible individuals, gut microbiota dysbiosis promotes disease onset and progression by disrupting immune regulation, impairing epithelial barrier function, and altering microbial metabolite profiles (Shan et al., 2022; Turner, 2009). Diagnosis traditionally requires multiple tests—such as blood work, computed tomography (CT) scans, and invasive procedures like gastroscopy, colonoscopy, and enteroscopy—placing considerable psychological and economic stress on patients (Braithwaite et al., 2021; Deding et al., 2023). In response, recent research has evaluated the diagnostic potential of fecal microbiota analysis, achieving an AUC of 0.966 in distinguishing patients with IBD from healthy individuals (Liang, 2021). Despite this, distinguishing CD from UC remains complex, with current studies primarily focused on bacterial profiles, often neglecting other microbiota components such as viruses, eukaryotes, and archaea.

This study addresses these gaps by integrating machine learning with traditional biological analyses. Machine learning, recognized for its capacity to manage large, intricate datasets and detect patterns without predetermined rules, has become instrumental in genomics and metagenomics, enhancing biomarker identification, outcome prediction, and diagnostic accuracy (Loomba et al., 2017).

Our approach includes k-mer analysis, which divides DNA sequences into subunits of length “k,” enabling the detection of subtle genetic variations within microbial communities (Koslicki and Falush, 2016). By leveraging k-mers extracted from fecal metagenomic data, our models effectively differentiate between CD, UC, and healthy controls, relying on gut microbiome data. This integration improves diagnostic accuracy and offers a less time-intensive, cost-effective alternative to conventional methods, supporting earlier, more individualized treatment strategies.

## Materials and methods

### Data source

**FIGURE 1:**
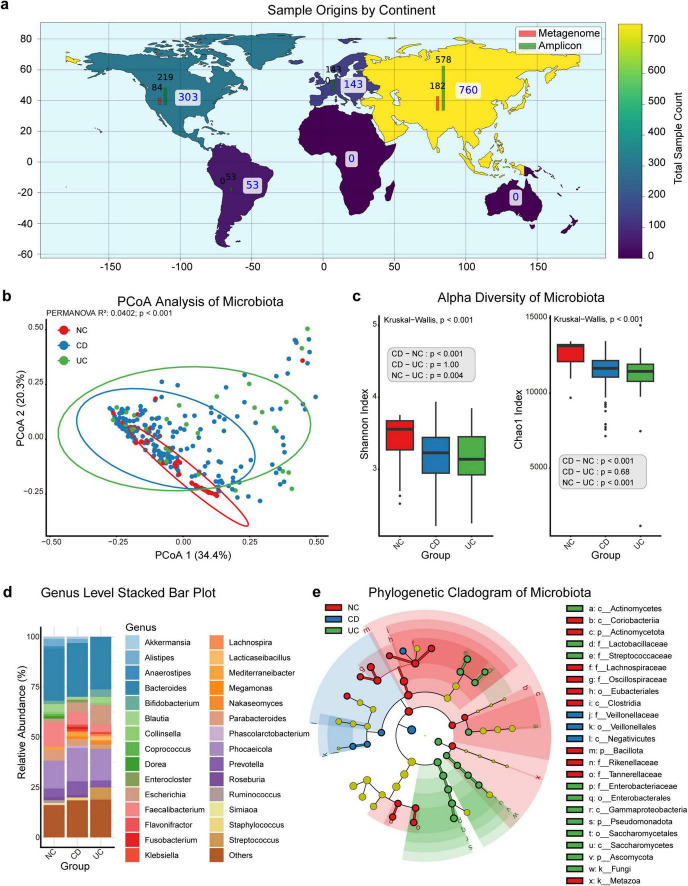
Microbiota distribution and diversity in NC, CD, and UC. **(a)** Global distribution of sample origins by continent, categorized by sequencing data type (metagenome and amplicon), with total sample count displayed using a color gradient. **(b)** Principal coordinates analysis (PCoA) plot illustrating microbiota clustering based on beta diversity, with groups including normal controls (NC), Crohn’s disease (CD), and ulcerative colitis (UC). **(c)** Box plots comparing alpha diversity of microbiota across NC, CD, and UC groups using the Shannon and Chao1 indices, with significant *p*-values from the Kruskal-Wallis test and *post-hoc* pairwise comparisons using Dunn’s test for each group. **(d)** Genus-level stacked bar plot showing the relative abundance of microbiota in NC, CD, and UC groups. **(e)** Phylogenetic cladogram displaying microbial composition across NC, CD, and UC groups. k, kingdom; p, phylum; c, class; o, order; f, family; g, genus; s, species.

Our research incorporates samples collected across multiple continents, with North America contributing 303 samples, consisting of 84 metagenome and 219 amplicon samples. South America provides 53 amplicon samples, without metagenome data. Europe contributes 143 samples, all of which are amplicon-based, while Asia supplies the highest total with 760 samples, comprising 182 metagenome and 578 amplicon samples (Figure 1a).

Sequencing data are accessible in the European Nucleotide Archive (ENA). Metagenome samples are derived from projects ERP017091, SRP075633, SRP324954, and SRP131166, while amplicon samples originate from ERP006859, SRP072280, SRP098586, SRP183770, SRP140631, SRP252115, SRP095645, SRP246037, SRP271847, and SRP131281. This study utilized data obtained from publicly available databases that provide anonymized and de-identified datasets. All procedures complied with the terms of use of these databases and relevant ethical guidelines. Therefore, ethical approval and informed consent were not required for this research.

Within the metagenome dataset, there are 39 normal control (NC) samples, 197 CD samples, and 30 UC samples (Supplementary Table S1). The amplicon dataset comprises 249 NC samples, 517 CD samples, and 227 UC samples (Supplementary Table S2).

### Raw data processing and quality control

Our previously published pipeline EasyMetagenome (Bai et al., 2025) was used to conduct microbiome analysis. Raw reads were processed with KneadData (v0.6.1), which (1) invoked Trimmomatic (Bolger et al., 2014) (v0.39) to remove adapters and trim low-quality bases, and (2) mapped the surviving reads to the GRCh37/hg19 human reference using Bowtie2 (Langmead and Salzberg, 2012) (v2.3.5.1). Reads aligning to the host genome were discarded. The resulting high-quality, host-depleted reads were subsequently forwarded to downstream taxonomic analysis.

### Taxonomic assignment of microbiome

Microbiome composition analysis was conducted with Kraken2 (Wood and Salzberg, 2014; Wood et al., 2019), aligning clean reads to the pluspf database, which encompasses sequences and annotations for bacteria, archaea, eukaryotes, and viruses, available at https://genome-idx.s3.amazonaws.com/k2_pluspf_20240605.tar.gz. The analysis applied default parameters to classify input reads, generating comprehensive reports with abundance data for each identified taxon. The resulting data were standardized using relative abundance metrics.

### Generating k-mer feature tables from high-throughput sequencing data

A feature extraction method was developed specifically for high-throughput sequencing data to efficiently extract k-mer features from FASTQ files, enabling rapid and precise analysis of large genomic datasets through parallel processing and accurate k-mer counting.

Initially, preprocessing was conducted with a custom script, GetKmerSignature.py, capable of processing both compressed and uncompressed FASTQ files and supporting batch reading from paired or single files. The script reads sequence data in 128-line batches per cycle, retaining only the base sequence lines for further analysis.

Next, a combinatorial method was used to generate all possible k-mer sequences composed of the four nucleotide bases (A, G, C, and T). This step employed a generator function that dynamically produces all k-mer combinations based on the user-defined length k, yielding 4*^k^* features. For this study, *k* values of 3, 5, and 7 were utilized for feature extraction.

In the feature extraction phase, k-mer frequencies within each sequence were calculated by first constructing a dictionary with k-mers as keys and their counts as values. Each sequence was then traversed to update the count of each relevant k-mer. Finally, k-mer frequencies in each sample were normalized by dividing each k-mer count by the total number of k-mers in that sample.

To optimize processing efficiency for large datasets, a parallel computing strategy was implemented using ProcessPoolExecutor, enabling concurrent processing of multiple samples and substantially improving computational speed. The results for all samples were consolidated and output to a designated temporary directory, with each output file containing k-mer frequencies and the sample name.

### Construction and evaluation of machine learning models

Four machine learning models—Logistic Regression (LR), Support Vector Machine (SVM), Naive Bayes (NB), and Feedforward Neural Network (FFNN)—were evaluated using 5-fold stratified cross-validation, which preserved class proportions across folds to ensure robust model assessment and mitigate the impact of data variability.

During each cross-validation fold, the training data was oversampled using the Synthetic Minority Over-sampling Technique (SMOTE) to address class imbalance, with augmentation restricted to the training subset. Features were standardized using a StandardScaler fitted on the training data, and the same scaling was applied to the validation data to maintain consistency. This standardization step enhanced model performance, accelerated convergence in gradient-based optimization, and ensured numerical stability. For the FFNN, data processing was conducted using TensorFlow (v2.13.1) and Keras (v2.13.1).

Model-specific configurations included setting the LR model (from scikit-learn) with a maximum of 1,000 iterations to ensure convergence. The SVM model employed a linear kernel with probability estimates enabled, while the NB model utilized GaussianNB. The FFNN architecture was configured with an input layer containing 16 units and ReLU activation, followed by a dropout layer with a rate of 0.5, a dense layer with 4 units and ReLU activation, and a final output layer with a single unit and sigmoid activation for binary classification. The FFNN was compiled with the Adam optimizer and binary cross-entropy loss function and trained for 50 epochs, incorporating early stopping with patience of 10 epochs to monitor validation loss.

An integrated model was developed to enhance predictive performance by combining the outputs of the four machine learning models—LR, SVM, NB, and FFNN—using a weighted voting approach. Each model’s contribution to the final prediction was weighted according to its ROC AUC score, which was scaled to assign a weight for each model. During each cross-validation fold, predictions from all models were aggregated, with the class receiving the highest weighted vote selected as the final prediction for each sample. This ensemble method utilized the strengths of individual models, offering greater accuracy and robustness than any single model alone.

To evaluate model performance, multiple metrics were employed, including ROC AUC, accuracy, F1 score, precision, recall (sensitivity), specificity, Matthews Correlation Coefficient (MCC), and Precision-Recall (PR) curves. Confusion matrices were generated for each fold and averaged across folds to provide a comprehensive view of classification performance. Model performance visualization was achieved by calculating and plotting the mean ROC curve across the 5 folds for each model, with standard deviation bands illustrating result variability.

### Data analysis and visualization

Data analysis and visualization were conducted using R (v 4.3.2) and Python (v 3.8). In R, data preprocessing utilized the packages dplyr, tidyr, data.table, and stringr, while microbial data analysis employed vegan, phyloseq (McMurdie and Holmes, 2013), and rstatix. Visualization in R was performed with ggplot2, ggpubr, pheatmap, and grid. Group comparisons for more than two groups were conducted using the Kruskal-Wallis test, with pairwise comparisons using Wilcoxon or Dunn’s tests. In Python, data preprocessing was managed using pandas, numpy (Harris et al., 2020), and scikit-learn. Class imbalance in microbial data was addressed with the imbalanced-learn library, applying the SMOTE technique to generate synthetic samples for minority classes. Feature selection was performed through Lasso regression, and dimensionality reduction was achieved using principal component analysis (PCA) from scikit-learn (Jolliffe and Cadima, 2016). Visualization involved matplotlib, with ROC curves and confusion matrices plotted to assess model performance. Model comparison metrics included sensitivity, specificity, recall, F1 score, accuracy, and AUC, with confidence intervals estimated *via* bootstrapping.

## Results

### Gut microbiota dysbiosis of patients with IBD

In this study, the diversity and composition of the gut microbiota—including bacteria, archaea, eukaryotes, and viruses—were analyzed across NC, CD, and UC groups. Principal Coordinates Analysis (PCoA) demonstrated clear clustering distinctions among these groups, indicating significant compositional differences in the gut microbiota (PERMANOVA, *p* < 0.001, Figure 1b).

Further analysis of alpha diversity using the Shannon Index showed significant intergroup variations (Kruskal-Wallis, *p* < 0.001, Figure 1c). The NC group displayed higher diversity than both the CD (Dunn’s Test, *p* < 0.001) and UC (Dunn’s Test, *p* = 0.0037) groups, while no significant difference was observed between the CD and UC groups (Dunn’s Test, *p* = 1.00). The Chao1 Index similarly indicated significant diversity differences (Kruskal-Wallis, *p* < 0.001), with the NC group again showing higher diversity than the CD (Dunn’s Test, *p* < 0.001) and UC (Dunn’s Test, *p* < 0.001) groups, and no significant diversity difference between CD and UC (Dunn’s Test, *p* = 0.68).

Genus-level analysis of gut microbiota composition across the NC, CD, and UC groups revealed significant enrichment and depletion of specific genera (Figure 1d; Supplementary Figure S1, Supplementary Table S3). The NC group was enriched with beneficial genera such as *Alistipes*, *Anaerostipes*, *Blautia*, *Collinsella*, *Coprococcus*, *Dorea*, *Faecalibacterium*, *Parabacteroides*, *Roseburia*, *Ruminococcus*, and *Simiaoa*, while *Escherichia* and *Klebsiella* were notably reduced. In the CD group, *Akkermansia* showed significant enrichment, potentially linked to probiotic use in CD treatment. Conversely, the UC group exhibited significant reductions in *Akkermansia*, *Alistipes*, *Phascolarctobacterium*, and *Staphylococcus*.

A phylogenetic cladogram was constructed to further examine microbial phylogenetic relationships (Figure 1e). This cladogram visualized taxa across multiple levels, highlighting distinct clusters for NC, CD, and UC groups, with taxa having a linear discriminant analysis (LDA) score above 4 included. The NC group was enriched with taxa including *Metazoa*, *Actinomycetota*, *Bacillota*, *Coriobacteriia*, *Clostridia*, *Eubacteriales*, *Lachnospiraceae*, *Oscillospiraceae*, *Rikenellaceae*, and *Tannerellaceae*. In the CD group, *Negativicutes*, *Veillonellales*, and *Veillonellaceae* were more abundant. The UC group was characterized by notable enrichment in *Fungi*, *Pseudomonadota*, *Ascomycota*, *Actinomycetes*, *Saccharomycetes*, *Gammaproteobacteria*, *Enterobacterales*, *Saccharomycetales*, *Lactobacillaceae*, *Streptococcaceae*, and *Enterobacteriaceae*.

These results underscore the complex, distinct microbial landscapes of the NC, CD, and UC groups, suggesting that microbiota composition differences may serve as biomarkers for differentiating disease states.

### Building diagnostic models using microbiota data

The gut microbiota plays a pivotal role in human health, with compositional dysbiosis closely associated with IBD. Distinct microbial signatures linked to IBD suggest that gut microbiota profiles could serve as effective non-invasive features in diagnostic models.

To assess the diagnostic potential of gut microbiota for IBD, four machine learning models—LR, SVM, NB, and FFNN—were constructed and evaluated using 5-fold cross-validation to ensure accuracy and generalizability. The outcome variable was simplified to two binary classifications: IBD vs. NC and CD vs. UC.

For both the NC vs. IBD and CD vs. UC classifications, LASSO regression was applied to select relevant features, and dimensionality was reduced to 16 dimensions using PCA for model construction (Supplementary Table S4). This process identified 23 features for the NC vs. IBD classification and 22 features for the CD vs. UC classification.

**FIGURE 2:**
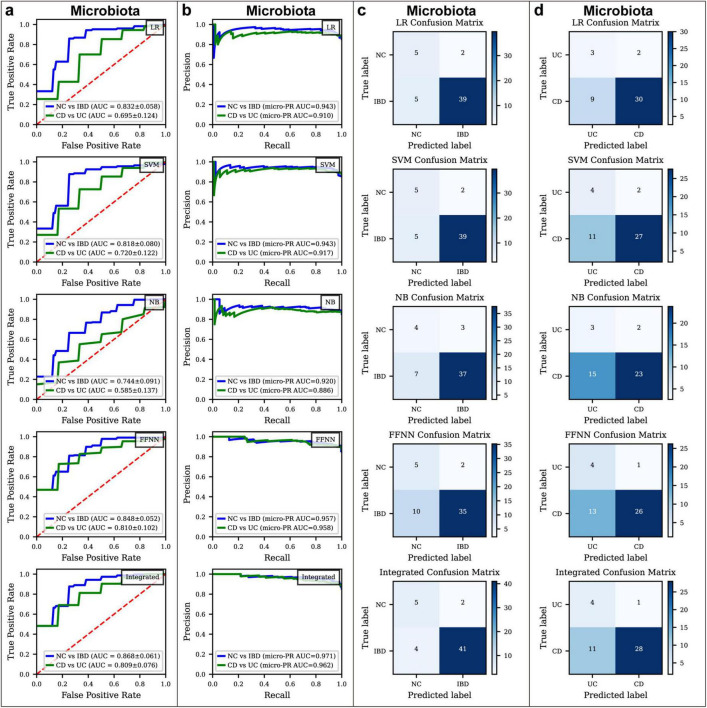
Classification performance of microbiota models in different disease status. **(a)** Receiver Operating Characteristic (ROC) curves for various classification models used to distinguish between normal control (NC) and inflammatory bowel disease (IBD), as well as Crohn’s disease (CD) and ulcerative colitis (UC), based on microbiota data. The models applied include logistic regression (LR), support vector machine (SVM), naïve Bayes (NB), feed-forward neural network (FFNN), and an integrated model. **(b)** Precision-Recall Curve for various classification models used to distinguish between NC and IBD, as well as CD and UC, based on microbiota data. The models applied include LR, SVM, NB, FFNN, and an integrated model. **(c)** Confusion matrices showing the performance of different models in distinguishing between NC and IBD based on microbiota data. **(d)** Confusion matrices showing the performance of different models in distinguishing between CD and UC based on microbiota data.

In the NC vs. IBD classification (Figures 2a,c; Supplementary Table S5), the FFNN model achieved the highest ROC AUC of 0.848, a Youden Index of 0.493, an MCC of 0.394, and a PR AUC of 0.966. The ROC AUC values for LR, SVM, and NB were 0.832, 0.818, and 0.744, respectively, with corresponding Youden Index values of 0.565, 0.565, and 0.392, MCC values of 0.503, 0.509, and 0.339, and PR AUC values of 0.949, 0.948, and 0.927. These results highlight the potential of microbiota-based models for effectively distinguishing IBD from healthy states.

In the CD vs. UC classification (Figures 2a,d; Supplementary Table S5), the FFNN model achieved a ROC AUC of 0.810 and a Youden Index of 0.465, an MCC of 0.324, and a PR AUC of 0.960. ROC AUC values for LR, SVM and NB were 0.695, 0.720 and 0.585, respectively, with Youden Index values of 0.328, 0.367, and 0.171, MCC values of 0.246, 0.263, and 0.119, and PR AUC values of 0.915, 0.921, and 0.885.

These findings underscore the challenge of distinguishing CD from UC based solely on gut microbiota, as the microbial differences between these conditions are subtle. To improve predictive accuracy, an integrated model was implemented using a weighted voting system, where each model’s vote was weighted by its respective ROC AUC score. This ensemble approach aimed to harness the strengths of the four models, designating a sample as positive if the weighted votes for the positive class surpassed those for the negative class. This method enhanced the reliability and robustness of our diagnostic tool, achieving an ROC AUC of 0.868 for distinguishing NC from IBD and an ROC AUC of 0.809 for differentiating CD from UC (Figure 2a), with Youden Index values of 0.621 and 0.411, MCC values of 0.563 and 0.293, and PR AUC values of 0.965 and 0.963 (Figures 2c,d; Supplementary Table S4), respectively, indicating significant improvements in predictive accuracy and robustness.

Despite these advancements, the diagnostic models for CD vs. UC differentiation fell short of expectations, with a ROC AUC of 0.809, Youden Index of 0.411, MCC values of 0.293, and PR AUC values of 0.963 (Figure 2; Supplementary Table S4). This relatively lower performance likely reflects the minimal microbial differences between CD and UC. To address these limitations, future work will explore new approaches, such as the k-mer method, which focuses on gene sequence variations. By capturing subtle genetic differences between CD and UC at the sequence level, the k-mer approach has the potential to uncover more informative features for model construction, ultimately enhancing diagnostic accuracy.

### Diagnostic potential of k-mer metagenomic data

To address the challenge of low AUC and Youden Index in models distinguishing between CD and UC, which fall below clinical standards, a novel approach was developed to significantly enhance these metrics. Traditionally, microbial abundance feature tables derived from metagenomic data require host sequence removal and classification. However, the absence of a comprehensive reference database results in considerable data loss. For instance, even advanced classifiers like Kraken2 leave 56% of reads unclassified when using the standard database (Hiseni et al., 2021). This unclassified microbial content, often referred to as “dark matter,” holds vast untapped potential.

To maximize data utilization, a bold approach was adopted by directly analyzing raw sequencing data without removing host sequences or performing classification. Instead, a sliding window (k-mer) approach was applied, calculating the frequency of each k-mer within DNA sequences obtained from sequencing. By leveraging the four nucleotides (ATCG), 4*^k^* unique k-mer features were generated and normalized to relative abundances, forming our final feature table.

This method circumvents traditional annotation limitations, allowing the direct use of gene fragments for diagnostic model construction, ensuring minimal data loss. Leveraging this comprehensive k-mer feature table enabled the development of more efficient diagnostic methods, reducing run time and enhancing throughput. These advancements not only improve processing speed but also enable more accurate and robust predictions.

Initially, diagnostic models were constructed using 3-mer, 5-mer, and 7-mer features, resulting in feature tables containing 64, 1,024, and 16,384 features, respectively. For each k-mer model, LASSO regression was applied to select relevant features, followed by PCA to reduce dimensionality to 16 dimensions for model construction. In the NC vs. IBD classification, 21, 22, and 22 features were selected for the 3-mer, 5-mer, and 7-mer models, respectively. For the CD vs. UC classification, 23, 19, and 24 features were selected for the respective k-mer models (Supplementary Table S4).

**FIGURE 3:**
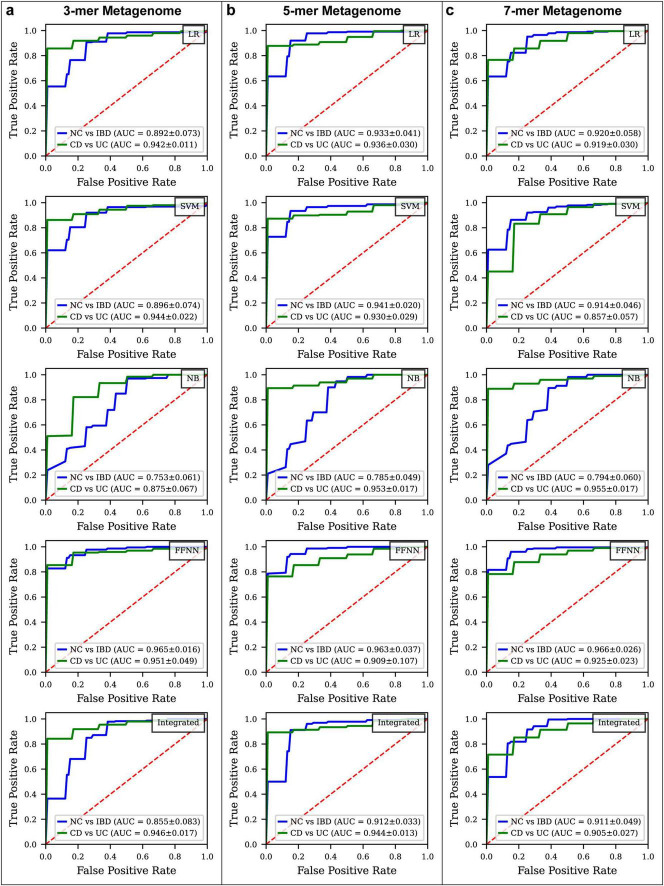
ROC curve comparison for different disease status classification models constructed By k-mer metagenome. **(a)** ROC curves for various models classifying IBD vs. NC and CD vs. UC using 3-mer metagenome data. **(b)** ROC curves for models using 5-mer metagenome data. **(c)** ROC curves for models using 7-mer metagenome data.

In the NC vs. IBD classification (Figure 3; Supplementary Figures S2, S4), the FFNN consistently yielded the highest ROC AUC across all k-mer models on the test set. For the 3-mer model, the FFNN achieved a ROC AUC of 0.965, Youden Index of 0.755, MCC values of 0.708, and PR AUC of 0.995. The ROC AUCs for LR, SVM, NB, and the integrated model were 0.892, 0.896, 0.753, and 0.855, with corresponding Youden Indexes of 0.691, 0.708, 0.438, and 0.662, MCC values of 0.617, 0.658, 0.363 and 0.597, and PR AUC values of 0.971, 0.976, 0.936, and 0.956 (Supplementary Table S6). In the 5-mer model, the FFNN reached a ROC AUC of 0.963 and a Youden Index of 0.718, MCC values of 0.697, and PR AUC of 0.992, while LR, SVM, NB, and the integrated models produced ROC AUCs of 0.933, 0.941, 0.785, and 0.912, with corresponding Youden Indexes of 0.745, 0.708, 0.444, and 0.684, MCC values of 0.658, 0.659, 0.354, and 0.635, and PR AUC values of 0.983, 0.989, 0.942, and 0.977 (Supplementary Table S7). For the 7-mer model, the FFNN recorded a ROC AUC of 0.966 and a Youden Index of 0.777, MCC values of 0.657, and PR AUC of 0.994, with LR, SVM, NB, and integrated models showing ROC AUCs of 0.920, 0.914, 0.794, and 0.911, and Youden Indexes of 0.707, 0.736, 0.282, and 0.664, MCC values of 0.622, 0.640, 0.205 and 0.520, and PR AUC values of 0.977, 0.977, 0.949, and 0.978 (Supplementary Table S8).

In the CD vs. UC classification (Figure 3; Supplementary Figures S3, S4), top-performing models varied based on the k-mer model. For the 3-mer model, the FFNN demonstrated excellent performance with a ROC AUC of 0.951 and a Youden Index of 0.868, MCC values of 0.769, and PR AUC of 0.992. The ROC AUCs for LR, SVM, NB, and the integrated model were 0.942, 0.944, 0.875, and 0.946, with Youden Indexes of 0.816, 0.793, 0.629, and 0.760, MCC values of 0.667, 0.662, 0.521, and 0.641, and PR AUC values of 0.991, 0.992, 0.965, and 0.992 (Supplementary Table S6). In the 5-mer model, the NB model achieved the highest ROC AUC of 0.953 and a Youden Index of 0.573, MCC values of 0.480, and PR AUC of 0.993, while LR, SVM, FFNN, and integrated models achieved ROC AUCs of 0.936, 0.930, 0.909, and 0.965, with Youden Indexes of 0.724, 0.701, 0.691, and 0.793, MCC values of 0.568, 0.562, 0.630, and 0.657, and PR AUC values of 0.990, 0.990, 0.979, and 0.992 (Supplementary Table S7). For the 7-mer model, the FFNN again delivered the highest performance with a ROC AUC of 0.925 and a Youden Index of 0.633, MCC values of 0.503, and PR AUC of 0.988, while LR, SVM, NB, and the integrated models had ROC AUCs of 0.919, 0.857, 0.955, and 0.905, with Youden Indexes of 0.620, 0.602, 0.326, and 0.593, MCC values of 0.499, 0.502, 0.244, and 0.425, and PR AUC values of 0.987, 0.969, 0.993, and 0.985 (Supplementary Table S8).

### Diagnostic potential of k-mer amplicon data

To address the high cost of metagenomic sequencing, which can impose a financial burden on patients, diagnostic models were constructed based on k-mer features derived from amplicon sequencing data.

Using this approach, this study developed models with 3-mer, 5-mer, and 7-mer features, applying LASSO regression for feature selection and PCA for dimensionality reduction (Supplementary Table S4). Specifically, for NC vs. IBD classification, 21, 19, and 19 features were selected for the 3-mer, 5-mer, and 7-mer models, respectively. For the CD vs. UC classification, 21, 17, and 33 features were selected for the corresponding k-mer models.

**FIGURE 4:**
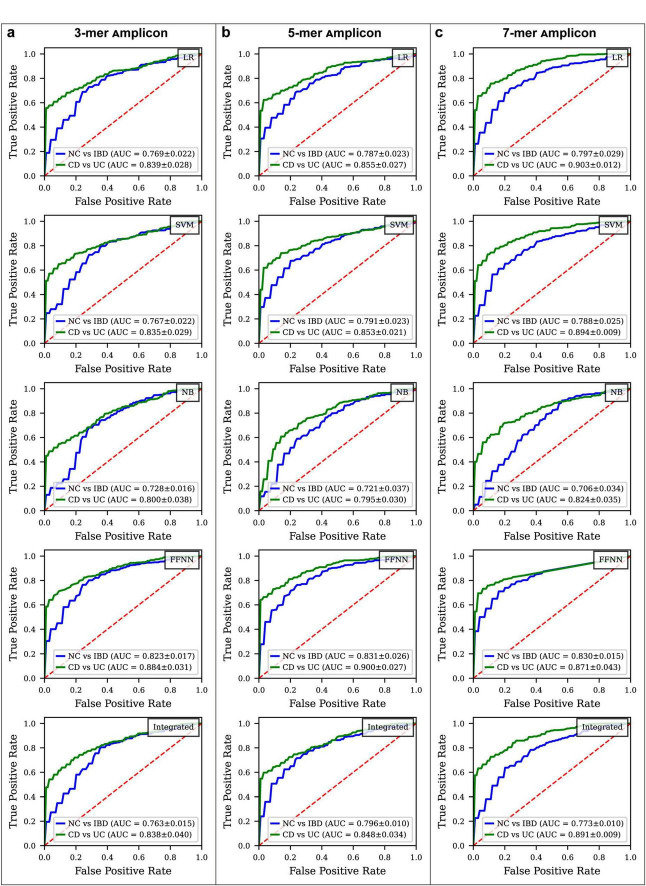
ROC curve comparison for different disease status classification models constructed by k-mer amplicon. **(a)** ROC curves for various models classifying IBD vs. NC and CD vs. UC using 3-mer amplicon data. **(b)** ROC curves for models using 5-mer amplicon data. **(c)** ROC curves for models using 7-mer amplicon data.

In the NC vs. IBD classification (Figure 4; Supplementary Figures S5, S7), the FFNN consistently achieved the highest ROC AUC across all k-mer models. For the 3-mer model, the FFNN attained a ROC AUC of 0.823 and a Youden Index of 0.466, MCC values of 0.390, and PR AUC of 0.956. The ROC AUCs for LR, SVM, NB, and the integrated model were 0.769, 0.767, 0.728, and 0.763, with Youden Index values of 0.390, 0.389, 0.350, and 0.414, MCC values of 0.308, 0.308, 0.267, and 0.336, and PR AUC values of 0.940, 0.939, 0.920, and 0.936, respectively (Supplementary Table S9). In the 5-mer model, the FFNN achieved a ROC AUC of 0.831 and a Youden Index of 0.476, MCC values of 0.414, and PR AUC of 0.958, while the LR, SVM, NB, and integrated models yielded ROC AUCs of 0.787, 0.791, 0.721, and 0.796, with Youden Indexes of 0.418, 0.438, 0.304, and 0.410, MCC values of 0.329, 0.344, 0.230 and 0.344, and PR AUC values of 0.949, 0.950, 0.918, and 0.950, respectively (Supplementary Table S10). For the 7-mer model, the FFNN reached a ROC AUC of 0.830 and a Youden Index of 0.529, MCC values of 0.413, and PR AUC of 0.962, whereas the LR, SVM, NB, and integrated models showed ROC AUCs of 0.797, 0.788, 0.706, and 0.773, with Youden Indexes of 0.494, 0.439, 0.264, and 0.418, MCC values of 0.395, 0.352, 0.208, and 0.346, and PR AUC values of 0.949, 0.945, 0.903, and 0.941, respectively (Supplementary Table S11).

In the CD vs. UC classification (Figure 4; Supplementary Figures S6, S7), the FFNN demonstrated the highest performance across all k-mer models. For the 3-mer model, the FFNN achieved a ROC AUC of 0.884 and a Youden Index of 0.601, MCC values of 0.545, and PR AUC of 0.955, while the ROC AUCs for LR, SVM, NB, and the integrated model were 0.839, 0.835, 0.800, and 0.838, with Youden Index values of 0.503, 0.521, 0.371, and 0.404, MCC values of 0.458, 0.476, 0.375 and 0.409, and PR AUC values of 0.938, 0.936, 0.918, and 0.935, respectively (Supplementary Table S9). In the 5-mer model, the FFNN achieved the highest performance with an ROC AUC of 0.900 and a Youden Index of 0.601, MCC values of 0.565, and PR AUC of 0.961. The LR, SVM, NB, and integrated models had ROC AUCs of 0.855, 0.853, 0.795, and 0.848, with Youden Indexes of 0.521, 0.556, 0.440, and 0.544, MCC values of 0.474, 0.506, 0.425 and 0.493, and PR AUC values of 0.944, 0.942, 0.899, and 0.941, respectively (Supplementary Table S10). For the 7-mer model, the LR demonstrated the best performance with a ROC AUC of 0.903 and a Youden Index of 0.622, MCC values of 0.567, and PR AUC of 0.960, whereas the SVM, NB, FFNN and integrated models attained ROC AUCs of 0.894, 0.824, 0.871, and 0.891, with Youden Indexes of 0.608, 0.455, 0.638, and 0.581, MCC values of 0.555, 0.441, 0.581, and 0.537, and PR AUC values of 0.957, 0.927, 0.954, and 0.957, respectively (Supplementary Table S11).

### 3-mer and 5-mer exhibit higher processing speeds compared to the traditional method

Processing times per gigabyte (GB) of data for feature table generation using 3-mer, 5-mer, and 7-mer methods were compared with the traditional Kneaddata + Kraken2 approach. Statistical analysis using the Kruskal-Wallis test, followed by Dunn’s test with Bonferroni correction for *post-hoc* analysis, revealed significant differences among the methods (*P* < 0.05).

The 3-mer method achieved a mean processing time of 456.37 ± 91.07 s/GB, significantly faster than the Kneaddata + Kraken2 method, which averaged 2130.03 ± 1626.63 s/GB (Dunn’s test, *p* < 0.05; Supplementary Figure S8). Similarly, the 5-mer method, averaging 1300.61 ± 376.85 s/GB, was significantly quicker than Kneaddata + Kraken2 (*P* < 0.05). In contrast, the 7-mer method had a mean processing time of 21,084.64 ± 7,873.43 s/GB, which was significantly slower than Kneaddata + Kraken2 (*P* < 0.05). These results suggest that while the 3-mer and 5-mer methods provide faster processing times than the traditional Kneaddata + Kraken2 approach, the 7-mer method demands substantially more time.

## Discussion

The analysis of gut microbiota diversity and composition in this study revealed significant differences between NC and patients with IBD, highlighting the potential involvement of gut microbiota in IBD pathogenesis and progression.

Significant clustering observed in PCoA indicates distinct microbial community structures among NC, CD, and UC groups. Consistent with previous findings (Clemente et al., 2018; Tian et al., 2024), higher microbial diversity was observed in healthy individuals compared to patients with IBD, reinforcing the association between microbial diversity and disease status and emphasizing the importance of considering geographic variability in microbiome studies.

Distinct microbial features identified across disease states facilitated the development of diagnostic models based on gut microbiota (Baxter et al., 2016; Baxter et al., 2016; Wirbel et al., 2019). While these microbiota-based models performed well in distinguishing NC from IBD, they were less effective at differentiating CD from UC. To address this, a k-mer-based approach was introduced to capture subtle genetic variations at the gene fragment level. This method demonstrated higher accuracy in differentiating CD from UC and outperformed traditional models in NC vs. IBD classification (Vervier et al., 2016).

Traditional microbiota analysis, which relies on microbial abundance feature tables, has notable limitations (Knight et al., 2018). Unannotated sequences or “dark matter” are often discarded, resulting in the loss of potentially valuable information (Iverson et al., 2012; Rinke et al., 2013). Additionally, reliance on reference databases requires significant computational resources, including memory and processing time, which limits clinical practicality (Quince et al., 2017; Sczyrba et al., 2017). Although these methods achieved strong ROC AUC scores for NC vs. IBD classification, their performance in CD vs. UC differentiation was comparatively poor.

In contrast, the k-mer method retains the entire sequencing dataset—including genomic “dark matter”—and therefore delivers a more comprehensive view of the microbiome. The fine-grained resolution afforded by k-mer profiling surpasses that of traditional pipelines, enabling the detection of subtle taxonomic and functional signals that illuminate novel metabolic pathways, microbial adaptations, and host-microbe interactions. Because k-mer analysis interrogates gene fragments directly, it pinpoints genetic distinctions between disease states and consistently yields higher ROC-AUC scores. Practically, shorter k-mers (e.g., 3-mers or 5-mers) generate feature tables rapidly, whereas the feature space for longer k-mers grows exponentially, markedly increasing computation time without appreciable gains in predictive power; thus, shorter k-mers provide the optimal balance between efficiency and performance for diagnostic modeling (Camarillo-Guerrero et al., 2021). Each microbial species possesses a unique genomic composition, so specific k-mers function as precise species-level fingerprints (Unal et al., 2023). Moreover, k-merspecies possesses a unique genomic composition, so specific k-mers function as precise species-level fingerprints omprehensive view of the mdeed, the discriminative k-mers we identified produced no significant BLASTN hits in the NCBI nt database, underscoring that our pipeline captures previously uncharacterized genomic fragmentspecies poss “dark matter” that conventional, reference-dependent methods would overlook—and demonstrating both the sensitivity and discovery potential of this approach.

A key observation in this study was the performance difference between metagenomic and amplicon sequencing data when using the k-mer method. Although metagenomic data achieved higher ROC AUC scores, this improvement came with longer processing times and a greater financial burden on patients. In our experience, metagenomic sequencing required substantially more computational resources and incurred notably higher costs compared to amplicon sequencing, potentially limiting its widespread clinical use. Balancing the benefits of enhanced diagnostic accuracy with the practical limitations of processing time and cost is essential. Stool-based tests are inherently convenient and non-invasive for patients, making them promising for large-scale screening and follow-up monitoring. By capturing a broader spectrum of microbial signals—including those not yet represented in existing reference databases (”microbial dark matter”)—the k-mer approach can potentially offer improved diagnostic coverage compared to traditional marker-based methods, which rely on prior knowledge of specific microbial sequences. Although shorter k-mers (such as 3-mers) may initially seem less specific, the collective pattern of these short fragments, when analyzed via modern computational and machine learning pipelines, can be highly discriminative. Moreover, the length of k-mers can be carefully selected to optimize both sensitivity and specificity for particular clinical applications. Nevertheless, the high-dimensional nature of k-mer data poses substantial computational challenges, and robust validation across diverse patient cohorts is needed before this method can be routinely integrated into clinical practice. We also acknowledge the importance of regulatory approval and cost-effectiveness studies to fully establish its feasibility and ensure broad accessibility in healthcare settings.

This study has several limitations, including sample size and the need for external validation. Future research should focus on validating these models in larger and more diverse cohorts to strengthen the evidence base and support the integration of these diagnostic tools into clinical practice. Additionally, longitudinal studies could provide insight into the models’ utility for monitoring disease progression or treatment response (Palladino et al., 2016).

In conclusion, while metagenomic sequencing remains the gold standard for microbiota analysis (Knight et al., 2018), its high cost and computational demands may restrict its feasibility in routine clinical settings. Amplicon sequencing offers a more accessible alternative but may compromise diagnostic accuracy. Selecting the appropriate sequencing method should consider clinical requirements, resource availability, and the desired balance between accuracy and feasibility. Tailoring the diagnostic approach to specific contexts ensures optimal patient care within practical constraints. These findings underscore the value of selecting suitable methodologies based on clinical needs, paving the way for advancements in microbiota diagnostics and contributing to more accurate, timely diagnosis of diseases like IBD (Chiu and Miller, 2019).

## Conclusion

This study highlights the promise of k-mer-based feature extraction methods in creating diagnostic models for IBD. This approach improves efficiency, fully leverages available data, and enhances model performance over traditional microbiota analysis techniques. However, overcoming the challenges posed by high-dimensional feature tables and further optimizing computational efficiency will be essential for successful clinical implementation. Our findings emphasize the potential of k-mer-based approaches, setting the stage for future innovations in microbiota diagnostics that can lead to more accurate and timely disease diagnoses, including for conditions such as IBD.

## Data Availability

Sequencing data are accessible in the European Nucleotide Archive (ENA). Metagenome samples are derived from projects ERP017091, SRP075633, SRP324954, and SRP131166, while amplicon samples originate from ERP006859, SRP072280, SRP098586, SRP183770, SRP140631, SRP252115, SRP095645, SRP246037, SRP271847, and SRP131281.
