# Supplementary figures and images for: Constructing inflammatory bowel disease diagnostic models based on k-mer and machine learning
Source: Front Microbiol. 2025 Jun 25;16:1578005. doi: 10.3389/fmicb.2025.1578005 (PMC12239758; doi:10.3389/fmicb.2025.1578005)

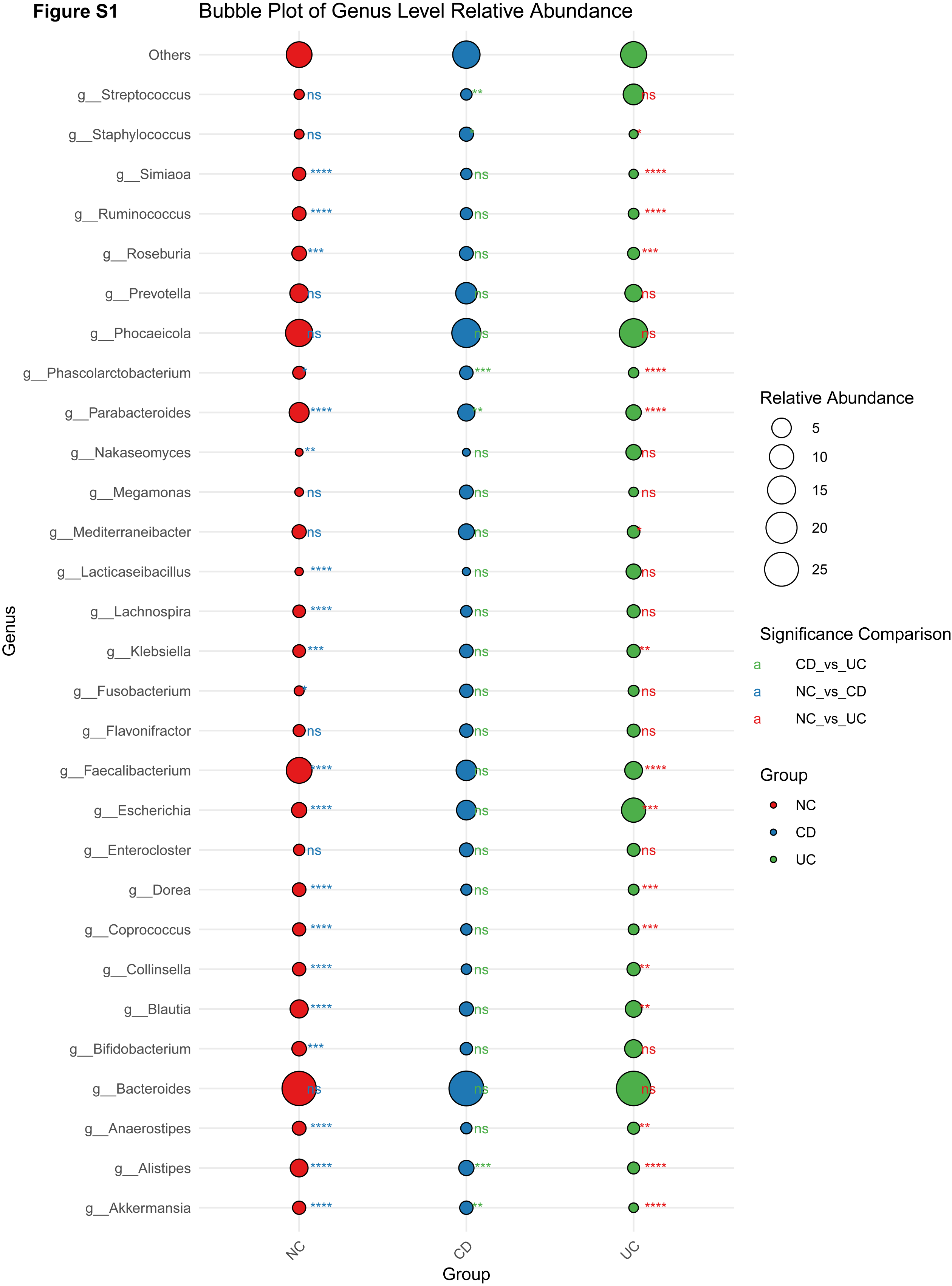

Supplement: Supplementary file 12 [file Image_1.tif]

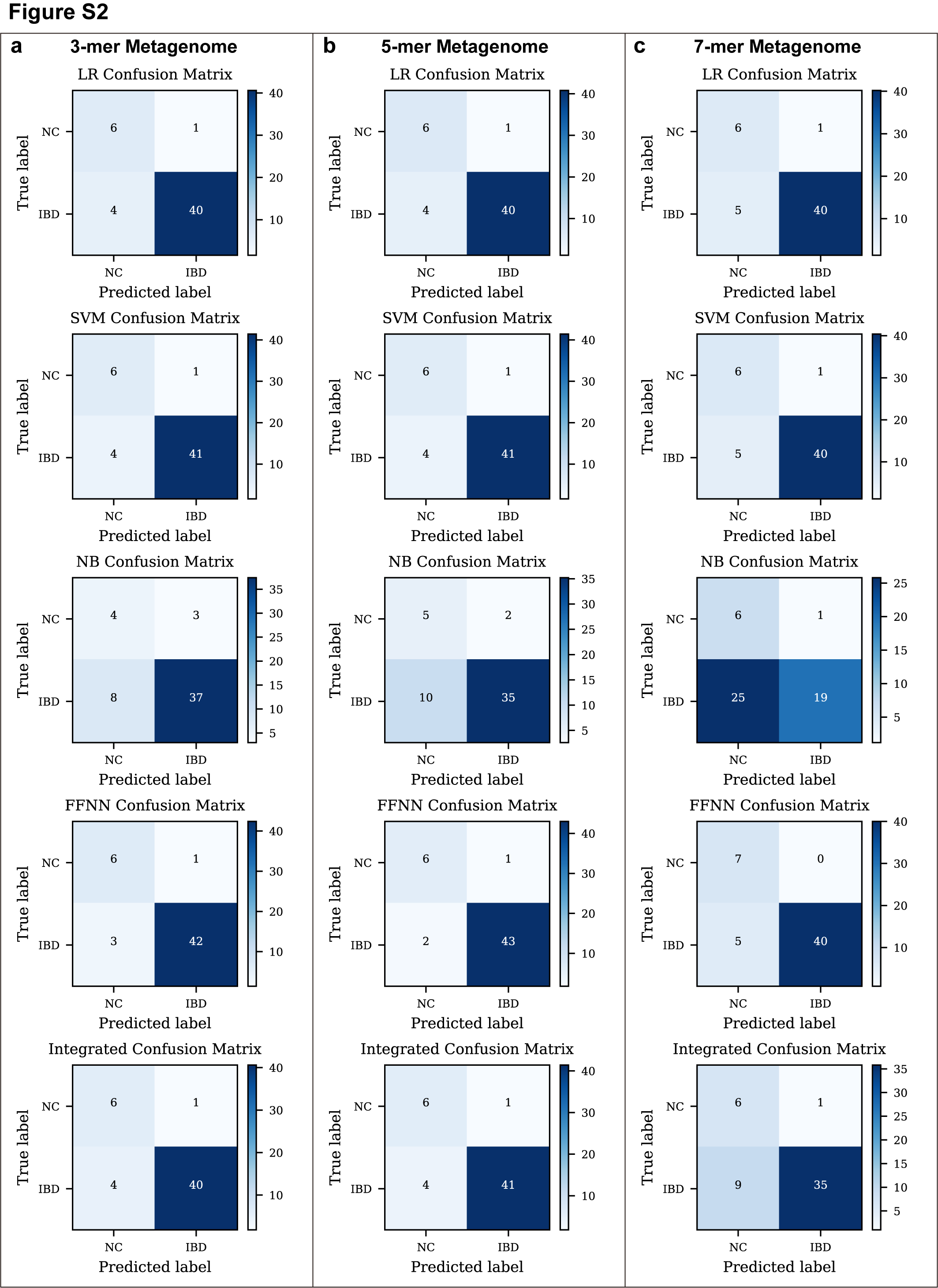

Supplement: Supplementary file 13 [file Image_2.tif]

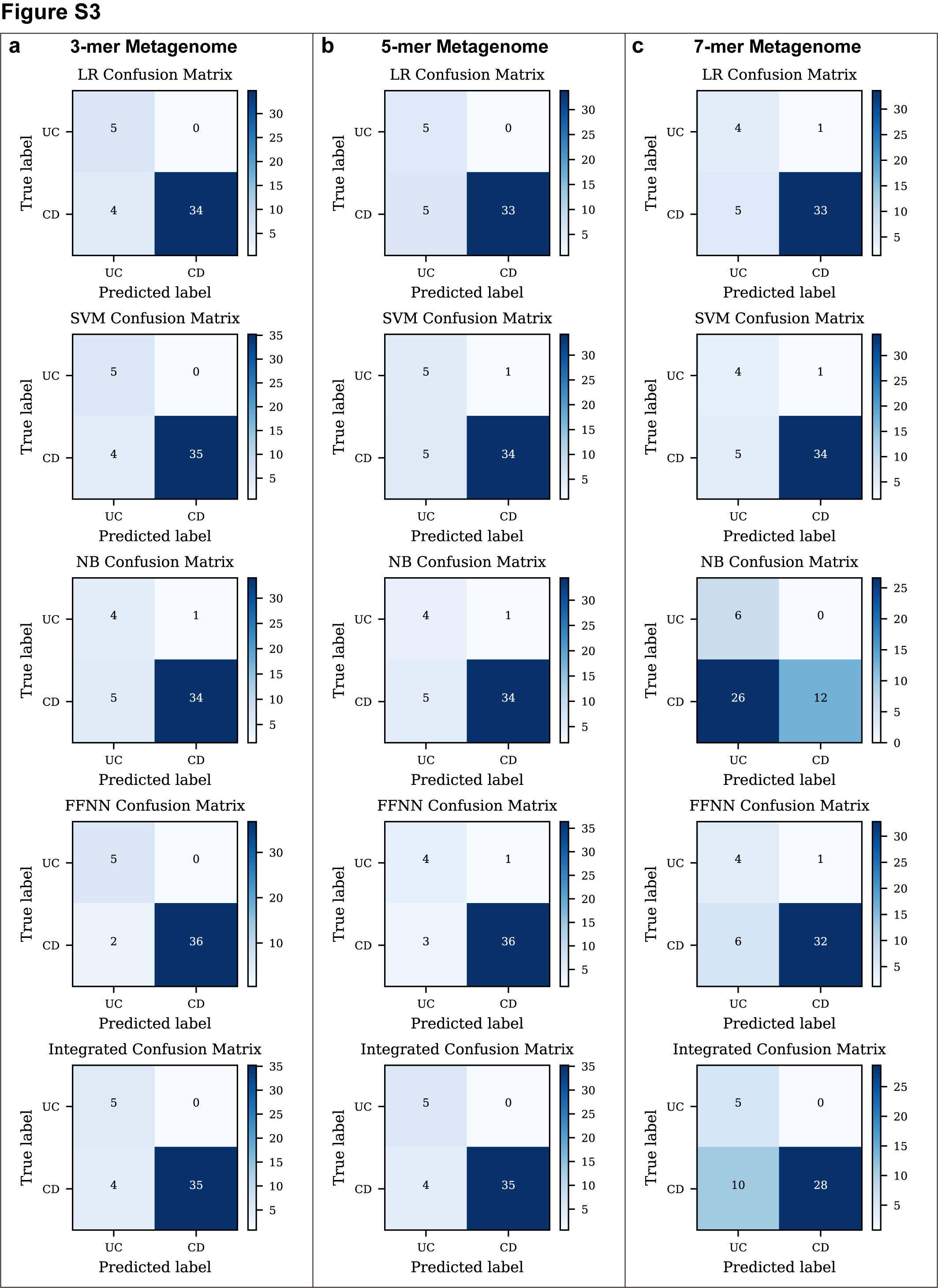

Supplement: Supplementary file 14 [file Image_3.tif]

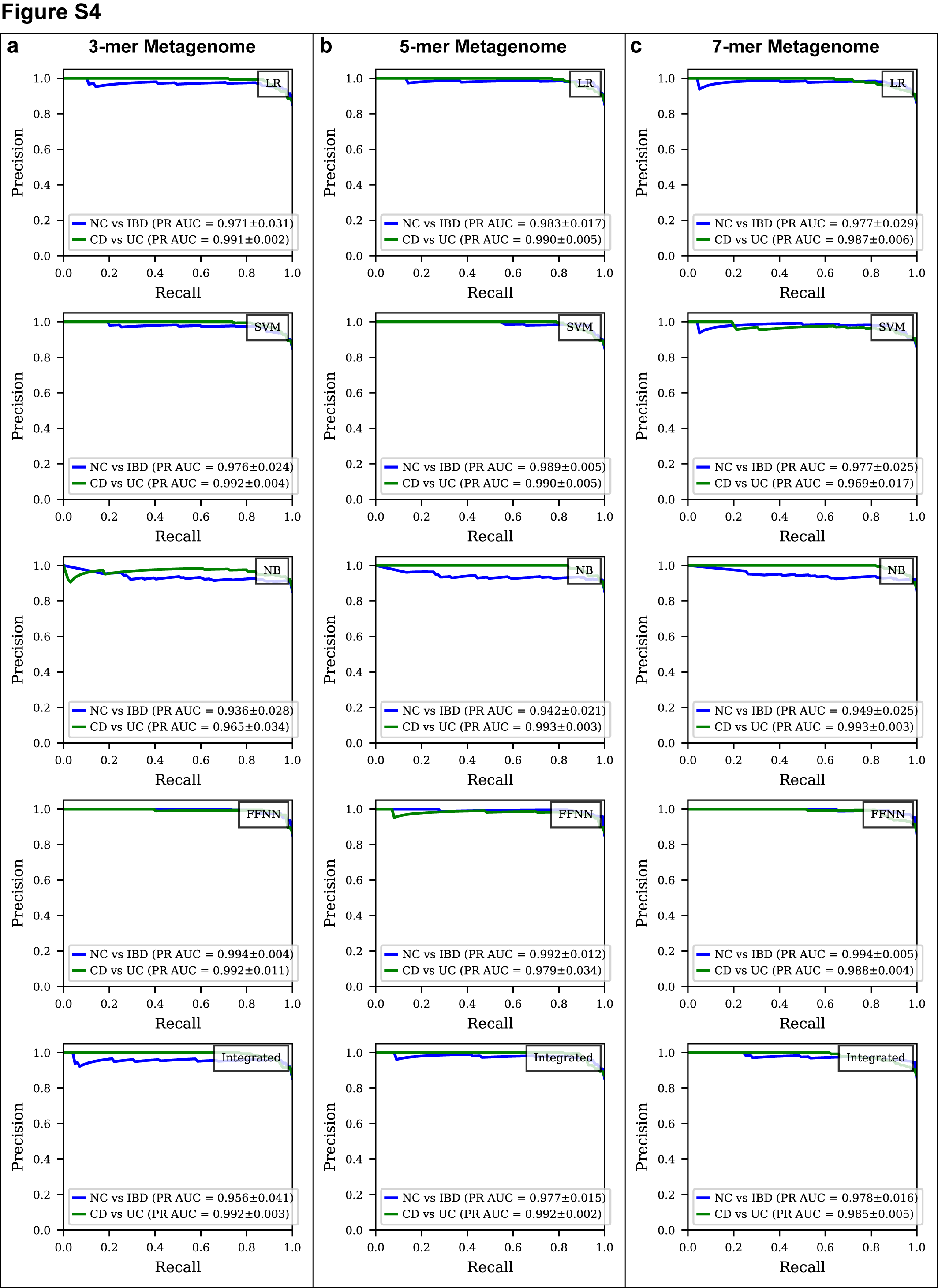

Supplement: Supplementary file 15 [file Image_4.tif]

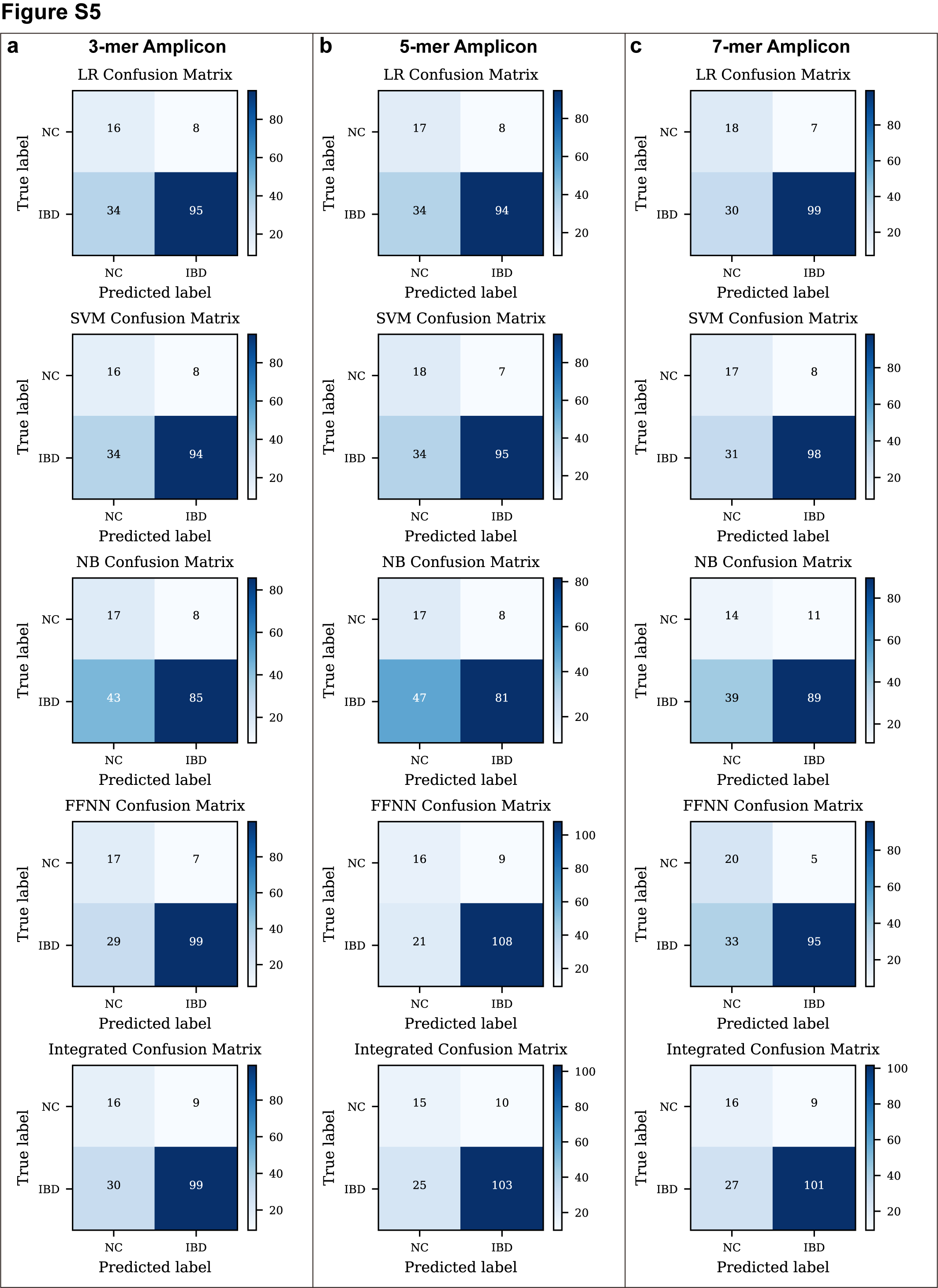

Supplement: Supplementary file 16 [file Image_5.tif]

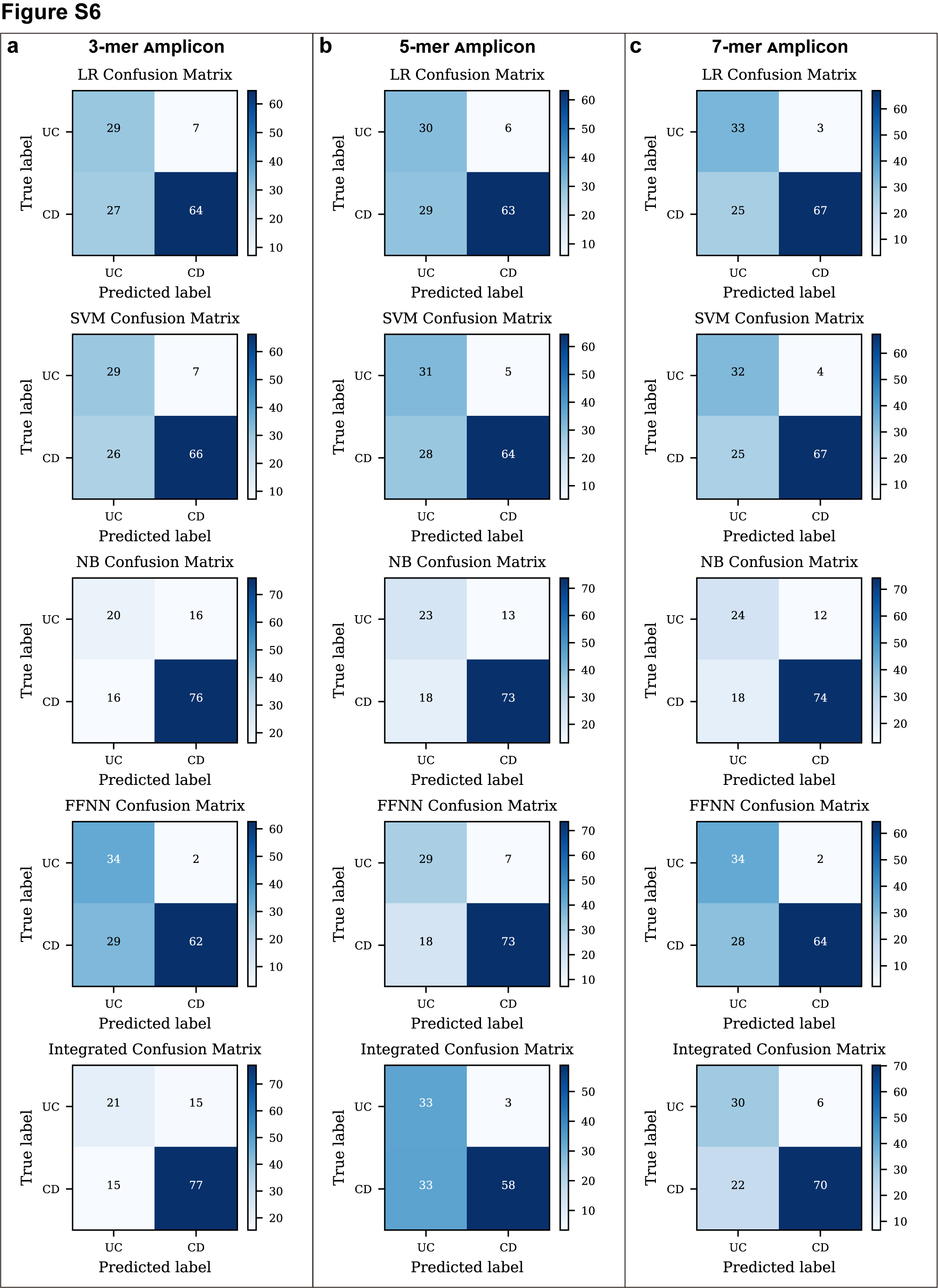

Supplement: Supplementary file 17 [file Image_6.tif]

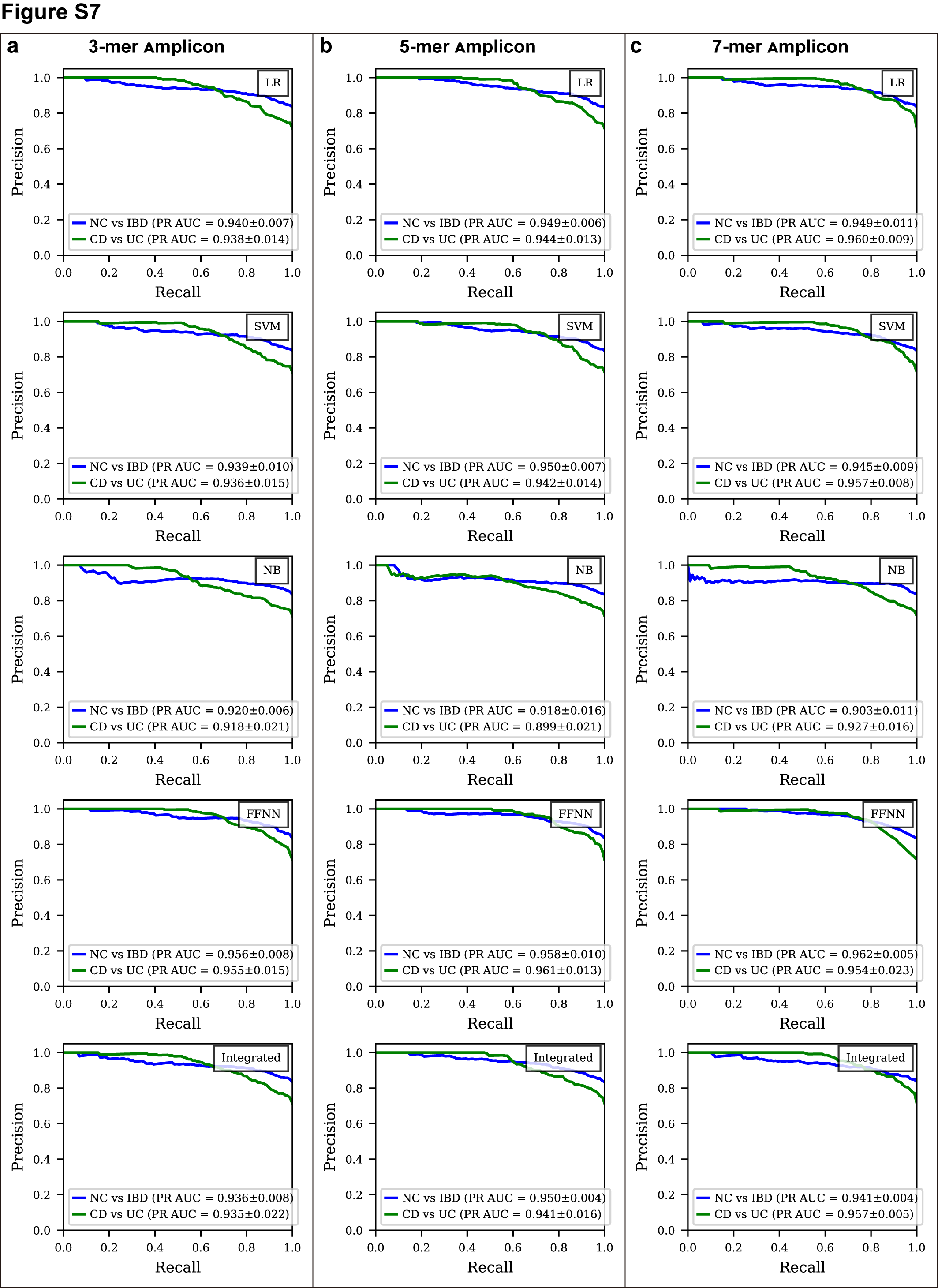

Supplement: Supplementary file 18 [file Image_7.tif]

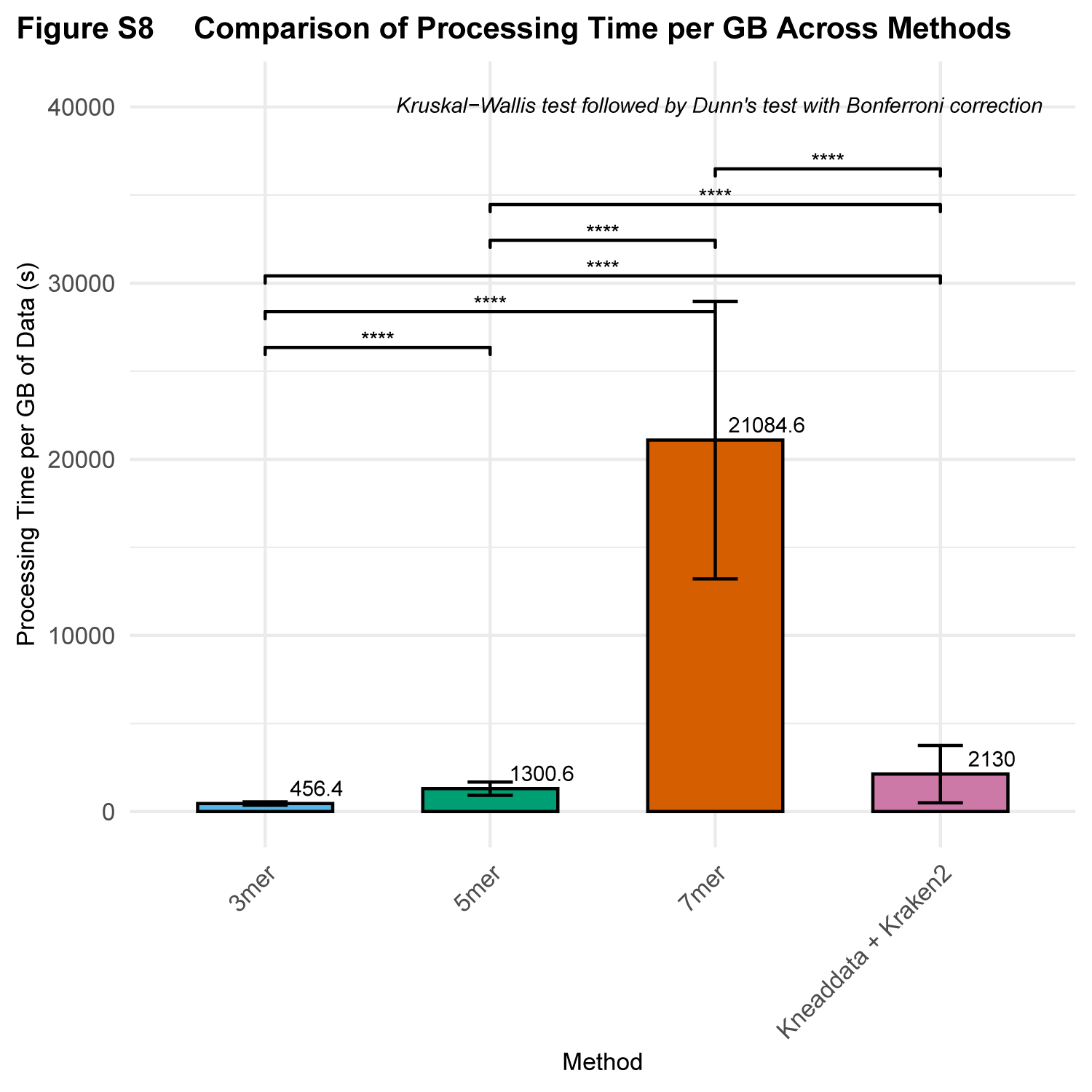

Supplement: Supplementary file 19 [file Image_8.tif]
